# Facilitating Return to Work through a Broader Perspective on Vocational Rehabilitation: Insights from Patients with Stress-Related Disorders

**DOI:** 10.1007/s10926-025-10311-5

**Published:** 2025-07-30

**Authors:** Anja Beno, Ingibjörg H. Jonsdottir, Agneta Lindegård

**Affiliations:** 1 Institute of Stress Medicine, Region Västra Götaland, Carl Skottsbergs Gata 22B, SE-413 19 Gothenburg, Sweden; 2 School of Public Health and Community Medicine, Institute of Medicine, Sahlgrenska Academy, Gothenburg University, Gothenburg, Sweden

**Keywords:** Exhaustion disorder, Return to work, Organisational changes, Management support, Individually tailored adjustments

## Abstract

**Background:**

Exhaustion disorder (ED) is a prevalent cause of sick leave in Sweden, and support from employers appears to facilitate return to work (RTW). The aim of this study was to explore and highlight what patients diagnosed with ED thought would have been beneficial for their RTW, on the individual level, on the workplace level and at the organisational level.

**Methods:**

Twenty patients were invited to participate in an interview conducted seven years after they sought care. The semi-structured interviews were transcribed verbatim and analysed using inductive content analysis.

**Results:**

The analysis revealed an overarching theme “A need for a holistic view of the RTW process” and three main categories emerged: “The importance of a well-prepared organisation”, “What characterises a good leader?” and “Meeting the needs of each employee”. Important findings were that adjustments are warranted on an organisational level. Leadership qualities such as having a supportive approach and authority to make changes were highlighted. On an individual level, influence on their work situation and tailored adjustments was essential. For most patients change of work situation, such as change of workplace, work tasks and reduced working hours were necessary.

**Conclusion:**

To facilitate RTW for patients with ED, it is essential to have a holistic approach that recognises necessary organisational changes, management support and individually tailored adjustments.

## Introduction

Mental illness and stress-related disorders has become one of the major challenges for modern society. An increasing number of individuals in the Swedish workforce are sick-listed due to stress-related diagnoses [1] and the same trend is seen all over Europe [2]. This leads to great costs, both on the individual level as well as on a societal level, with expenses both in social insurance [3] and in productivity loss [4]. Decreased work participation due to stress-related disorders can be a consequence of many different negative factors connected to both the private sphere [5] as well as to unfavourable working conditions [6]. However, previous research has identified poor working conditions as a major risk of being diagnosed with stress-related disorders [7–9]. Previous studies found several work-related factors associated with stress-related diagnoses, such as high demands, low job control, high workload, low reward, job insecurity, deficient leadership, high effort–reward imbalance, low co-worker support, low procedural and relational justice [8–11].

In Sweden the clinical diagnose stress-related exhaustion disorder (ED) was introduced into clinical practice in 2005 [12]. The main symptoms of ED are lack of physical and mental energy, cognitive dysfunction, sleep disturbance, reduced stress tolerance and somatic symptoms [13, 14]. Patients with ED show similar symptoms as individuals assessed to have burnout [15] and these symptoms have in earlier research shown to negatively influence perceived work productivity [16].

Several methods such as cognitive behaviour therapy (CBT), graded physical activity and activity regulation are used for treatment of symptoms of ED [17, 18]. Even though cognitive behavioural therapy (CBT) seems to reduce symptoms, none of these treatments/interventions have been effective in decreasing sick leave duration or enhance early return to work (RTW) [19–23]. Moreover, studies indicate that many people with ED are on sick leave and for some of them it takes years for full return to work [24, 25]. However, there are some studies indicating that earlier RTW can be obtained for this patient group if the employer’s is involved early in the rehabilitation process [26–29].

The research on predictors for RTW for patients with common mental disorder, including ED, describe a complex mixture of different factors. Successful RTW for patients with episodes of poor mental health can be predicted by factors related to work, social factors and medical conditions [30]. Few studies have focused on the patient’s own experiences and desires during the RTW process, Norlund et al. found that both the patient’s own resources and practical/emotional support from the employer were important for successful RTW [31]. In a qualitative study by Stromback et al. where rehab coordinators used a dialogue-based workplace intervention, participants described that communication and collaboration improved relationships, knowledge sharing and attitudes towards ED amongst supervisors and colleagues and also enhanced opportunities for RTW [32]. For some patients with ED, full RTW could take several years. In a recent study, we found that 93% of patients with ED had returned to work after 7 years; however, the majority of these patients made some kind of change at work due to their disease, such as change of workplace, work tasks or working hours [33]. Thus, changes at work could be necessary for some patients with ED, but the aforementioned study also raises the question of how patients experienced the RTW process and what kind of arrangements facilitate RTW and sustainable work life over the long term.

The aim of this study was to explore how patients diagnosed with ED experienced the RTW process, including what they thought would have been beneficial for RTW on the individual, workplace and organisational levels.

## Methods

### Study Design

An inductive qualitative design was used to explore participants’ perceptions of factors associated with successful RTW. The present study was part of a larger follow-up study described by Glise et al. [25].

### Participants

All patients included in this study were admitted to the Institute of Stress medicine (ISM), a specialist tertiary clinic in the region of Västra Götaland, Sweden. At this clinic, patients fulfilling the criteria for ED received treatment between 2004 and 2016. At the clinic, patients were provided with personalised treatment consisting of similar components tailored to their specific needs. Patients had regular visits with a physician, and consultations with a psychologist or other psychotherapist were offered if needed. The importance of lifestyle changes, including physical activity and sleep, was consistently emphasised during clinic visits. Physiotherapists provided individualised guidance on graded physical activity. Those experiencing severe sleep problems were offered cognitive behavioural group therapy towards the end of their rehabilitation process. Psychoeducation and stress reduction programmes were available to all patients following their initial consultation [34].

Patients eligible for follow-up with a follow-up period of at least 7 years and no more than 12 years (n = 353) were invited to meet with a physician at the clinic, and a total of 136 (46%) patients agreed to participate. This procedure is described by Glise et al. [14]. In connection with this clinical assessment, the patients were asked to participate in a qualitative interview about their work situation during their work-related rehabilitation process. All patients had returned to work as they were included in the study. Patients were consecutively invited to participate in the interview study, with the aim of achieving variation in gender, age, educational level and work sector, until 20 participants had provided their consent. Characteristics of the participants are described in Table 1.

**Table 1:** Characteristics of the participants

Characteristics	Women (n = 14)	Men (n = 6)	Total (n = 20)
Age			
Mean (range)	50 (32–60)	45 (37–51)	49 (32–60)
Education			
Lower	3 (21%)	0 (0%)	3 (15%)
Higher	11 (79%)	6 (100%)	17 (85%)
Employment			
Public sector	9 (64%)	2 (33%)	11 (55%)
Private sector	5 (36%)	4 (66%)	9 (45%)

Lower education: elementary school/upper secondary school.

Higher education: university education.

### Interviews

Semi-structured interviews were conducted to explore participants’ RTW process over the preceding seven-year period. The interviews were performed by a qualified ergonomist at the specialist clinic and lasted between 30 and 45 min. The interview guide was designed to use open-ended and non-directive questions to be able to allow in-depth dialog with the patient about their experience. Consisting of four main questions, which were consistently applied across all interviews, the process sought to gain insights into participants’ perceptions of potential work adjustments due to their illness, their experience of the rehabilitation and return-to-work (RTW) process and the factors they identified as critical for successful rehabilitation outcomes. The main interview questions are described in Box 1.

Box 1: Example of interview questions Questions.

If your working life has changed during the past seven years, can you describe the changes (change of work tasks, workplace or change of working hours).

If you made changes at work, in what way were the changes important for you to be able to work the way you want to?

In your particular case, what actions at the workplace do you think have been the most important to the outcome of your rehabilitation?

What do you think is important at the workplace to ensure a sustainable RTW for patients with ED?.

### Analysis Method

The interviews were analysed using qualitative content analysis with an inductive approach, enabling the visualisation of both manifest and latent content [35–37].

The last author of this paper performed all the interviews and also worked during the study as a ergonomist at the research institute conducting the study. The first author worked as a physician and PhD student at the same institute. The interviews were transcribed verbatim by another independent researcher. In the first step, all interviews were read and analysed separately by the first and the last author, annotations were made in the margin to highlight meaning units corresponding to the aim of the study. In the second step, the first and last author individually formed codes from the meaning units. In the third step, the first and last author together merged the individually formed codes into sub-categories and categories. The process is depicted in Table 2. The same procedure was repeated for all interviews. Finally, the first and last author formed an overarching theme reflecting the latent content of the empiric data.

**Table 2:** Example of the analysis process

Meaning unit	Condensed meaning unit	Code	Subcategory	Category
“Well, it´s like this, during the rehabilitation process, you put a lot of resources on the individual but do nothing about the workplace”	Focus only on the individual during the rehabilitation process and not on the workplace	From individual to workplace focus	Do organisations want to see the “bigger picture”?	The importance of a well-prepared organisation
"You need a manager who can actually remove tasks or reorganise things. Otherwise, it’s just talk".	Manager who can actually remove or organise things	Practical authority needed to make changes	Can managers really enable changes?	What characterises a good leader?

#### Ethics

The study was conducted in compliance with the Declaration of Helsinki and was approved by the Regional Ethical Review Board in Gothenburg (ref: 668–15). All participants provided informed consent before the interview.

## Results

The primary objective of this study was to explore the factors that participants perceived as important for a successful RTW. The analysis resulted in a main theme, “A need for a holistic view of the RTW process” encompassing all the categories and sub-categories. This overarching theme is a summary of the latent content embedded within our analyses, functioning as an umbrella construct that integrates all identified categories and sub-categories. Furthermore, it aligns closely with the overarching aim of the study, to elucidate the aspects participants perceived as important during the RTW process. Three main categories were formed “The importance of a well-prepared organisation”, “What characterises a good leader?” and “Meeting the needs of each employee”, each with 2–3 sub-categories (Table 3).

**Table 3:** Main theme, categories and sub-categories associated with successful RTW

Main theme	A need for a holistic view of the RTW process						
Categories	The importance of a well-prepared organisation			What characterises a good leader?		Meeting the needs of each employee	
Sub-categories	Do organisations want to see the “bigger picture”?	Providing structure in work organisation	Fostering a culture of awareness and dialogue	A need for multifaceted and present leadership	Can managers really enable changes?	Ensuring employees influence in the RTW process	Adjustments in working conditions

## Main Category 1: The Importance of a Well-Prepared Organisation

Organisational prerequisites for implementing changes and adjustments in the workplace were found to be important for RTW. Moreover, increased knowledge amongst managers and human resources (HR) about stress-related exhaustion, clear goals and good communication within the organisation were also mentioned as key factors that enable successful RTW.

### *Do organisations Want to see the “Bigger Picture”?*

Participants in the study highlighted the significance of the work environment throughout the RTW process, with a focus on both the broader organisational perspective and the group level within the workplace. Some participants noted that attention is frequently directed towards the individual level, rather than addressing issues across multiple levels within the organisation. The rehabilitation process often emphasises individual-level adjustments, such as coping strategies, rather than examining the workplace setting itself.

“If you clean a staircase, you start at the top. Otherwise, the crap will be left”.

According to the participants, many colleagues at the workplace faced similar issues, but the approach was to make individual adjustments rather than addressing and resolving the problems at the organisational level, such as improving structures, processes, culture or working conditions across the entire organisation.

### *Providing Structure in Work Organisation*

The participants emphasised the importance of workplace structure in facilitating successful RTW. This included having a broader understanding of the objectives of the workplace, well-defined tasks and a clear understanding of the individual’s role and responsibilities. The participants highlighted the need for a structured and well-defined environment with clear expectations of employees, the department and the organisation as a whole.

“There is a goal, and you can see it all along, and I can work towards that goal, and I know there will eventually be an end”.

Other important factors were having a clear assignment description or well-defined work tasks. Some participants expressed a desire for more specific job responsibilities and not being obligated to perform tasks for which they lack the proper education or expertise.

### *Fostering a Culture of *Awareness* and Dialogue*

Several factors in the contextual work environment were identified as important during the rehabilitation process, such as communication about adjustments at different levels in the workplace, both between managers and employees and higher up in the organisation. In order to create a greater understanding across the organisation, participants also noted the importance of knowledge about stress-related mental health problems and its consequences amongst managers, HR and colleagues. The sense of community and team spirit in the workplace is important, and some participants described the importance of feeling welcomed and secure at the workplace.

“… you can have communication with your manager, and you can have communication with your co-workers… I think it is an important key factor”.

A prevailing theme amongst the participants was the perception that there was inadequate knowledge in the organisation regarding the causes and consequences of ED in the workplace. This was described as a lack of awareness regarding stressful work conditions (e.g. reorganisations), workplace conflicts, overtime, ambiguous job descriptions and deficient feedback and communication between managers and employees. Additionally, many participants highlighted the need for greater understanding of how ED affects work capacity and the significance of implementing workplace adjustments to facilitate successful RTW.

“It´s important to prevent it from happening in the first place. Take better care of people and don’t push them to break down. Don’t assume that you should deliver and that you are smart because you work around the clock. This will have consequences. If you know that in advance, it might not have to happen”.

Some of the participants said that the most important thing was that the manager and HR had knowledge about stress-related mental health problems and how they can affect the ability to work. In the context of the RTW process, it was highlighted that providing information to colleagues in the workplace is crucial. This information should include an understanding of stress-related mental health problems, associated symptoms that can impact a person’s ability to work and the need for workplace accommodations.

Several participants emphasised the importance of acceptance amongst colleagues and managers during the RTW process. This could mean ensuring that the individual feels included in the group, despite being unable to perform at the same level, and allowing the individual to ask for help without fear of being degraded or judged as less capable. Participants described that feeling comfortable and being able to speak their mind in the workplace were important factors in the RTW process. Additionally, feeling included in a workgroup was important for RTW. Several participants also pointed to the importance of a sense of fellowship and the ability to have enjoyable moments together.

### Main Category 2: What Characterises a Good Leader

Many of the participants highlighted the importance of the qualities of the leadership during the RTW process. Key factors mentioned included active involvement and presence in the daily work of the organisation, demonstrating empathy, being a reliable stakeholder throughout the RTW process and ensuring the provision of essential adjustments.

### *A Need for Multifaceted and Present Leadership*

Several participants identified various leadership qualities as essential, including being present and engaged, showing empathy and building a trusting relationship with the employee.

Participants highlighted that it is important for managers to be present in the day-to-day work. This helps managers gain a better understanding of different work tasks and responsibilities, which in turn can aid the evaluation of the employee’s work ability and performance.

“I think that what is important is that a good employer has a, so to speak, better understanding and feeling to find ways to evaluate one’s ability earlier”.

Having a better understanding and being present in different everyday work situations can facilitate the early detection of issues that may not be apparent at first glance, according to several participants. Attentiveness amongst the leadership team was also described as way to facilitate the ability to identify the employee’s strengths and limitations and to adapt work tasks to maximise their job performance.

Participants emphasised the need for empathetic leadership. During sick leave, leadership may need to maintain regular contact with the employee and show that they care. Empathetic communication with the employee makes the RTW process easier and reduces pressure.

“He called me while I was on sick leave, asked ‘how you are’. How are you? The most important question was, ‘How are you?’ It’s kind of human to ask"

Additionally, some participants stated that it was important for managers to express that the most important thing was for the employee to recover, and that the manager showed patience during the RTW process. Several participants mentioned the significance of building a trusting relationship between the manager and the employee. This involves having confidence in the manager and feeling trusted in return. According to some of the participants, a trusting relationship must be mutual.

### *Can Managers Really Enable Changes?*

Many participants highlighted the importance of managers being responsive to employees’ needs and making necessary adjustments without making employees feel inadequate. Managers need to clearly communicate that these adjustments do not affect their perception of the employees’ abilities.

“When it has been stressful – because it has been for me sometimes…Then I have told her (my manager). And then she says ‘Yes, then we will remove it immediately.’ So, then she says… ‘And this will not affect my opinion of you XX’”.

Identifying and managing conflicts or tensions amongst colleagues was also mentioned to be essential, as unresolved interpersonal issues affects return to work. Participants underlined that it is the manager’s responsibility to recognise and handle such situations during the RTW process. It is also necessary to identify contradictory or conflicting situations amongst colleagues and ensure that the manager takes responsibility for resolving these issues. Facilitating adjustments is essential, and the manager has a crucial role in implementing these adjustments. Many participants stated that they initially needed additional time to return to work with a decreased workload.

Several participants highlighted the need for the manager’s assistance in choosing particular work tasks.

“It´s crucial, that you feel that you get support. It´s difficult to pick work tasks when you get back. Knowing that you have support to avoid specific situations or people, or whatever is most difficult. I think that is important no matter what the cause is”.

Certain work tasks can be more demanding and challenging, and it can be beneficial for the individual if these work tasks are either entirely removed or kept to a minimum.

## Main Category 3: Meeting the Needs of Each Employee

Responsiveness to individual preferences refers to the ability or willingness to adjust and respond to the individual’s needs and wishes during the RTW process. Several factors were recognised as important, including the ability for the individual to control (autonomy) which work settings he or she would be required to work in. Another crucial element was a well-defined plan and individual adjustments authorised by the employer.

### *Ensuring Employees Influence in the RTW Process*

Most participants described influence as a key factor for a successful RTW, including the ability to make decisions about when and where to work, to control the pace of work and to influence which work tasks will be performed.

“Being able to control your working hours a little, so that it is more flexible”.

Being in a stressful situation due to time constraints and deadlines was mentioned as a difficult, and making adjustments to prevent this during the RTW process was found to be beneficial.

Having a workspace with fewer distractions was considered essential by several participants. They expressed the need for a private room at work or to transfer to another department whilst retaining their current workload.

“I work in an open office space, and we will not change that...It’s like sitting at a train station. And I am very easily disturbed by a lot of noise and people around me… mmm, and now I have a slightly smaller room with fewer people, and it is more of a corner, which feels important”.

Participants highlighted the importance of having the option to work from home. This was partly because it provided a quieter and less distracting work environment, but also because it saved time and energy that would otherwise be spent commuting. The ability to choose what worked best for them was considered a crucial factor. Some participants found it important to have easier work tasks and a clear ending to work tasks, that is, to be able to “cross off a list” and finish their duties. The ability to remove “unnecessary work tasks” and focus more on their actual work duties was also described as important. Having clear goals and a clear understanding of what was expected in relation to their profession increased the ability of participants to create a sense of control and set their own parameters, so they could achieve their goals.

Most participants highlighted the importance of having influence at work. For some of them, influence meant having the freedom to make decisions.

“…but my job is also allowing a great freedom and – how do you say it – freedom with responsibility”.

Participants highlighted the importance of an individualised rehabilitation plan that takes into account both medical and work-related needs. They described that having influence over the structure of their RTW process, such as starting with part time work, gradually increasing working hours and receiving tailored support based on their needs at different stages was crucial for ensuring a sustainable RTW.

### *Adjustments in Working Conditions*

The participants were asked about changes at work, that is, a change of workplace, work tasks or working hours. The majority of the participants reported that they had changed workplace, a few of them several times. All participants described that their work tasks changed to some extent, regardless of whether they had stayed at the same workplace or changed workplace. For many of the participants, temporary changes were made during the rehabilitation process, fore some participants the changes were permanent. However, some participants made the necessary changes a few years after they had returned to full-time work. Some participants described the change as an opportunity to choose a different path.

“This is one of the insights I made during this exhaustion. The way to become better is that you can make a new start. You can choose another path”.

Changing workplace was sometimes crucial, both to be able to recover from exhaustion and regain work capacity. Some participants described that they had to make more than one change in the last few years to find a good work environment. A few changed their career or position, but most participants remained in the same profession.

“I´ve changed jobs three times before I ended up where I am today and decided what I want to do, and I feel very good in my present work situation”.

A few participants reduced their working hours due to their illness to be able to RTW and remain at work, but a few also reported that they were able to work more hours than before because they made certain changes and adjustments at work.

## Discussion

The main findings of this study include a range of participant-identified factors that were perceived as important for successful RTW across individual, workplace and organisational levels. Based on the manifest content, these were summarised in the categories “The importance of a well-prepared organisation”, “What characterize a good leader” and “Meeting the needs of each employee”. In addition, a latent theme emerged from the interviews, pointing to the importance of taking a broader, holistic view of the entire rehabilitation and RTW process.

Participants emphasised the importance of a structured and well-defined work environment, with clear job expectations and flexible work schedules, to support a successful return to work. This is in line with previous research by Villott et al. (2021), who found that similar organisational factors were associated with successful RTW for individuals with musculoskeletal disorders and common mental disorders [38]. Despite the knowledge that organisational interventions are crucial for successful RTW, a recent review found that most intervention studies are still directed towards the individual [39]. Employers need to have a clear understanding of their role and responsibilities with respect to the RTW process. It has been shown that having appropriate job tasks that matched one´s skills and expertise was essential for a sustainable RTW for patients with mental disorders [39]. Furthermore, the participants in our study also pointed out that the ability to have part-time or modified work schedules during the RTW process would have facilitated their rehabilitation process. The importance of workplace support both from employers and colleagues, together with clear communication between employers and employees, was also highlighted as important. Thus, our findings are well in line with previous research [19, 29, 40, 41].

A sense of community and a welcoming atmosphere at the workplace were also found to be beneficial, with participants describing the importance of feeling “secure” at work. These findings are also consistent with results from an earlier study by Pijpker et al. [42]. According to our participants, a smooth return-to-work process is often hindered by the lack of knowledge and awareness about stress-related illnesses amongst managers, HR representatives and co-workers.

A similar study by Lysaght et al. found that social support in the form of trust, effective communication and an understanding of how disability influences job performance were crucial for a successful RTW [43].

In the context of the RTW process participants highlighted the importance of specific leadership qualities. These valued leadership characteristics include accessibility, being considerate, being understanding, being empathetic and being appreciative. These findings are in line with previous studies where participants emphasised the need for managers to be present and engaged in day-to-day work, to show empathy and build a trusting relationship with employees [44–48]. However, the authority of managers and ability to make organisational changes differs considerably depending on the work organisation and the context in which the work tasks are performed. Bjork et al. found that managers in feminised organisational contexts face less favourable organisational conditions compared to managers in masculinised organisations [49]. An analysis of potential differences between sectors was not possible due to the narrow scope of this qualitative study, but differences in the conditions encountered by managers in different sectors should be explored in future studies.

An important finding in the present study was that participants emphasised the importance of having influence in the RTW process, including the ability to decide when and where to work, and to regulate work pace and tasks. The results reveal that workplaces need to be more flexible to improve the RTW process for patients with ED. The balance between job demands and job control was also highlighted as important during the RTW process. A good work-life balance is widely known to have a positive impact on work-related stress [50]. Participants also emphasised the importance of a concrete, individualised and closely monitored rehabilitation plan with gradual RTW.

Changes of work situation, such as relocating to a different workplace, adjusting work tasks, or changing working hours were often perceived to be necessary to facilitate a successful return to work. Previous research on workplace changes is scarce for this patient group; however, in a previous study, we found that over a 7-year period, almost two thirds of patients with ED made changes at work [33]. In the present study, these changes were not always made immediately after the return to work; on the contrary, participants needed time to determine which changes were needed to create a sustainable work life. This highlights the importance of a gradual RTW programme that can be incorporated into an individualised rehabilitation plan. The findings also suggest that changes in the workplace can be an important factor for a successful RTW process, as it can provide a better work environment for the individual. Rooman et al. found that a change in employer was a factor that facilitated RTW [51]. Similar findings were also reported by Boštjančič et al., where participants found it easier to make changes in a new workplace [52]. For some individuals who worked part time before sick leave, it was also possible to work more hours than before due to certain changes and adjustments at work. On the other hand, some participants found that a reduction in working hours was crucial for successful RTW.

### Strengths and Limitations

A strength of the study was that the study was conducted after an extended period, giving the participants the opportunity to reflect on the process and gain perspectives beyond those reported immediately after RTW. Another potential strength of the study was that the researchers had different backgrounds, where one author has a stronger medical background and the other focuses more on rehabilitation.

The study sample was biased towards women as they were overrepresented in the sample. Gender distribution was not specifically managed during recruitment, and hence, the sample reflects the gender distribution of ED patients treated at the clinic, which may limit the transferability of the findings to men with ED. Furthermore, patients treated at the specialist clinic generally had a higher level of education than the general population, and referral bias may have existed, as this group may have sought specialist care from their general practitioners to a greater extent. Additionally, it is possible that the study is biased towards patients who were willing to participate, such as those who had not fully recovered and wanted to reconnect with the clinic, or those who had a positive treatment outcome and wished to contribute to the research. To mitigate this potential bias, the study team relied on their knowledge of the patient group and validated the results through dialog with the clinical team to ensure the applicability of the results to a larger patient sample. Due to the convenience sampling strategy, the findings may not represent the full range of RTW experiences. Furthermore, recall bias must be considered as a limitation since the interviews were performed 7–10 years after the first visit to the clinic. Another limitation of the study is that the interview guide could have been more specifically tailored to the aim, which may have limited the exploration of latent content. However, the interviewer’s attentive and responsive approach—through relevant follow-up questions and allowing participants to elaborate—helped to elicit deeper insights despite this limitation.

## Conclusion

This study contributes to a better understanding of the rehabilitation and return to work process for patients with stress-related exhaustion, highlighting the importance of adopting a holistic view in this process. We conclude that patients with ED emphasised the need for more specific workplace adjustments at both individual and organisational levels, together with adequate prerequisites that enable managers to support a sustainable return to work.

## Data Availability

No datasets were generated or analysed during the current study.
